# Challenging Diagnosis of Myocardial Infarction Due to Anomalous Left Circumflex Artery

**DOI:** 10.5935/abc.20180093

**Published:** 2018-07

**Authors:** Maksymilian P. Opolski, Kajetan Grodecki, Mateusz Spiewak, Mariusz Furmanek, Ilona Michalowska

**Affiliations:** 1 Institute of Cardiology, Warsaw - Poland; 2 Medical University of Warsaw, Warsaw - Poland; 3 Medical Center for Postgraduate Education, Warsaw - Poland

**Keywords:** Myocardial Infarction / Diagnosis, Coronary Artery Anomalies, Coronary Angiography, Cardiac Magnetic Resonance

**Figure 1:**
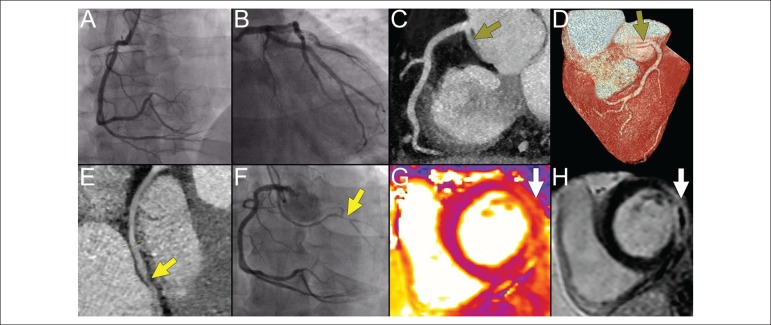
Coronary angiography, coronary computed tomography angiography and cardiac magnetic resonance findings of the patient with challenging diagnosis of myocardial infarction and anomalous left circumflex artery.

A 45-year-old male without past medical history presented with retrosternal chest pain and ST-segment elevation in inferolateral leads at ECG. Invasive coronary angiography, along with optical coherence tomography performed as part of the clinical study, showed normal coronaries, and myocardial infarction with non-obstructive coronary arteries (MINOCA) was diagnosed (Figure 1 A-B). Due to ongoing chest pain, triple-rule-out computed tomography angiography (CTA) was undertaken to exclude aortic dissection and pulmonary embolism. Incidentally, anomalous left circumflex artery (LCx) originating from the right sinus of Valsalva with a suspicion on severe stenosis was detected (Figure 1 C-E). Selective angiography of the LCx confirmed severe lesion in the distal vessel segment (Figure 1 F), however given the resolution of patient's symptoms, a decision on medical therapy with dual antiplatelet agents was undertaken. At discharge, cardiac magnetic resonance disclosed mildly reduced left ventricular ejection fraction (53%) with myocardial edema and transmural infarction of the basal-to-mid lateral wall (Figure 1 G-H).

LCx arising from the right aortic sinus is the most frequent coronary artery anomaly (CAA) found in up to 0.7% of the population. Although anomalous LCx is considered benign, the severe angle and tortuous vessel course may predispose it to accelerated atherosclerosis. Herein, the anomalous LCx was overlooked due to super-selective cannulation of the right coronary artery, and a large intermediate branch was incorrectly classified as LCx leading to deferred revascularization and irreversible myocardial injury. This case highlights that CAA could be included in the differential diagnosis of MINOCA, and unveils the potential for triple-rule-out CTA in detecting CAA.

